# Effectiveness of Dry Needling versus Placebo on Gait Performance, Spasticity, Electromyographic Activity, Pain, Range-of-Movement and Quality of Life in Patients with Multiple Sclerosis: A Randomized Controlled Trial Protocol

**DOI:** 10.3390/brainsci10120997

**Published:** 2020-12-17

**Authors:** Carlos Luque-Moreno, Anabel Granja-Domínguez, Jose A. Moral-Munoz, Guillermo Izquierdo-Ayuso, David Lucena-Anton, Alberto Marcos Heredia-Rizo

**Affiliations:** 1 Department of Nursing and Physiotherapy, University of Cadiz, 11009 Cadiz, Spain; carlos.luque@uca.es (C.L.-M.); david.lucena@uca.es (D.L.-A.); 2 DINAC Foundation, Vithas Nisa Hospital, 41950 Seville, Spain; anabel.granja@fundaciondinac.com (A.G.-D.); guillermo.izquierdo@fundaciondinac.com (G.I.-A.); 3 Institute of Research and Innovation in Biomedical Sciences of the Province of Cadiz (INiBICA), University of Cadiz, 11009 Cadiz, Spain; 4 Departamento de Fisioterapia, Facultad de Enfermería, Fisioterapia y Podología, Universidad de Sevilla, 41009 Sevilla, Spain; amheredia@us.es

**Keywords:** multiple sclerosis, gait disorders, neurologic, pain management, muscle spasticity, physical therapy modalities, dry needling, clinical trial protocol

## Abstract

Dry needling (DN) is an emerging technique commonly used in neurological and musculoskeletal pain conditions, but there have been no previous studies in patients with multiple sclerosis (pwMS). This trial aims to assess the efficacy of deep DN, compared with sham placebo DN, on gait performance, spasticity level, pain, electromyographic activity, range-of-movement (ROM) and quality of life in pwMS. Forty adults with MS were randomly assigned to one study group. The DN group will undergo 2 sessions (once per week) using DN over the rectus femoris (RF) and gastrocnemius medialis (GM) muscles at the lower extremity with higher spasticity. The placebo group will receive the same protocol using a sham placebo needle (Dong Bang needle). Outcome measures will include gait performance, using the GaitRite^®^ system, spasticity level with the Modified Ashworth Scale, superficial electromyographic activity of RF and GM, pain (pressure algometer), ROM (goniometer), and quality of life (Musiqol). This study is the first investigating the short-term effect of DN, compared with placebo, in pwMS, and taking into account the possible changes in the electromyographic activity of the lower limb. Therefore, the results may help to understand the suitability of using this technique in the clinical setting for this population. Trial registration: ACTRN12619000880145.

## 1. Introduction

Multiple sclerosis (MS) is one of the most common and disabling neurologic conditions among early adults, affecting approximately 2.3 million people worldwide [1], and with a higher prevalence in women (3:1 ratio) [2]. The specific cause of the disease is still uncertain, and thus the individual course and prognosis are difficult to predict [3]. Persons with MS (pwMS) suffer from varying cognitive and sensory-motor impairments that affect their functional capacity, including impaired gait performance [4]. This is reported as a major complaint in up to 85% of pwMS [5], and seems to be closely related to increased fall risk [6]. Chronic pain, especially at the lower extremities (LE), is also a very disabling symptom within this population, with an overall prevalence of 50% [7]. Spasticity and muscle weakness are present from the initial stages of the disease and have been associated with abnormal gait patterns [8], which may worsen other symptoms and impact on the quality of life (QOL). For example, the presence of spasticity contributes to modifying the speed, cadence, stride length, and swing phase during gait, and to a lower hip and knee range-of-movement (ROM) [9]. Additionally, walking-related motor fatigue in pwMS is attributed to the higher proportion of force that LE muscles, especially the soleus and gastrocnemius muscles, have to generate during prolonged walking [10].

The clinical management of MS-related disorders includes pharmacological and non-pharmacological interventions. In severely disabled patients, rehabilitation aims to rebalance the altered metabolic and muscle parameters [11], and the main goal is to improve functional independence and QOL [12]. Overall, physiotherapy treatments can be categorized into four domains: physical training (fitness, endurance, or resistance training), neuroproprioceptive facilitation or inhibition, motor skill acquisition, also called guided individualized therapy, and technology-based interventions [13]. However, the evidence for most approaches is still limited for the management of aspects such as walking capability [12], spasticity level [14], and self-reported pain intensity [15,16]. In addition, the recommendations about the necessity of improving the access to treatment for pwMS, to adapt the interventions to the individual disability level, and to use up-to-date evidence-based approaches do not seem to be properly implemented in the clinical setting [17,18].

Dry needling (DN) is a novel but commonly used technique for patients with musculoskeletal pain, although there is conflicting evidence about its effectiveness [19,20]. In people with central nervous system disorders, the efficacy of DN has been scarcely investigated in stroke survivors, with purported positive effects on spasticity, pain, and ROM [21,22,23]. Even though DN is mostly a safe technique, minor adverse events, e.g., bruising, bleeding, and pain, are common [24], and need to be considered.

The specific effect of DN, when compared with sham placebo DN, has been barely assessed in people with neurological conditions [25]. Patients’ expectations regarding the treatment may influence the results and evoke changes associated with the placebo effect. In fact, it has been suggested that the patient–practitioner relationship may be enough to improve the QOL and the results after intervention in some patients [26], although there is certain discrepancy about this topic [27]. In chronic musculoskeletal disorders, there are no definite conclusions on the superiority of using real deep DN over placebo DN [28]. Therefore, more studies that compare these two approaches are needed to better understand this issue.

To our knowledge, no previous randomized controlled trials have investigated the effects of using deep DN in pwMS. Recently, our research group reported promising findings in a clinical case study following the same protocol [29]. DN aims to induce a localized stretch of the soft tissue and may help to modulate the motoneuron activity and decrease the excitability of spinal reflexes, even though the exact physiological mechanisms that support this technique remain unknown [30]. The use of DN also seems to optimize the results of rehabilitation protocols. For example, in people with fibromyalgia, DN has been proposed as a suitable complementary approach to improve pain and QOL [31,32]. However, it is still necessary to continue evaluating the potential clinical effects of DN in many other conditions [30]. We hypothesize that using deep DN over the rectus femoris (RF) and Gastrocnemius Medialis (GM) muscles, which are directly related to the functionality of the LE, may help to evoke motor control changes that improve gait performance in pwMS. Therefore, our aim is to analyze the short-term effects of deep DN, compared to sham placebo DN, on gait parameters, spasticity level, electromyographic activity, pain intensity, ROM, and QOL in adults with MS.

## 2. Materials and Methods

### 2.1. Study Design

This is a protocol for a placebo-controlled, double-blind, superiority, randomized clinical trial. The study protocols follows the guidelines of the SPIRIT Statement [33]. This study protocol was approved by the Andalusian Biomedical Research Ethics Committee 04/2019 (CEI Hospitales Universitarios Virgen Macarena—Virgen del Rocío, Seville, Spain) and was registered on the Australian New Zealand Clinical Trials Registry (ACTRN12619000880145).

### 2.2. Setting and Eligibility Criteria

The data will be collected in the Multiple Sclerosis Research and Treatment Unit, Dinac Foundation, Vithas Nisa Hospital, Castilleja de la Cuesta, Seville, Spain. Eligibility criteria are as follows: patients with a diagnosis of MS according to the McDonald 2010 criteria [34]; males and females older than 18 years; ability to walk for at least 2 min (with or without support), which represents an Expanded Disability Status Scale (EDSS) [35] score between 0–6.5; absence of MS outbreaks in the previous month; having signed the informed consent form; and being able to comply with the study protocol. Exclusion criteria are: pregnancy; patients with comorbidity signs in the last month; having received corticosteroid treatment within the previous month; inability to walk due to physical impairments; and having received botulinum toxin injections at the LE in the last 3 months.

### 2.3. Procedure

**Figure 1:**
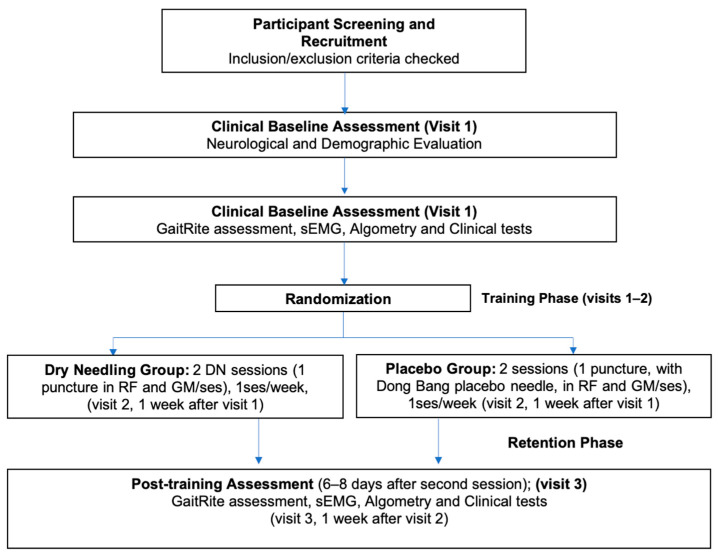
Study design flowchart. GM: Gastrocnemius medialis; RF: Rectus femoris; sEMG: surface Electromyography.

**Figure 2:**
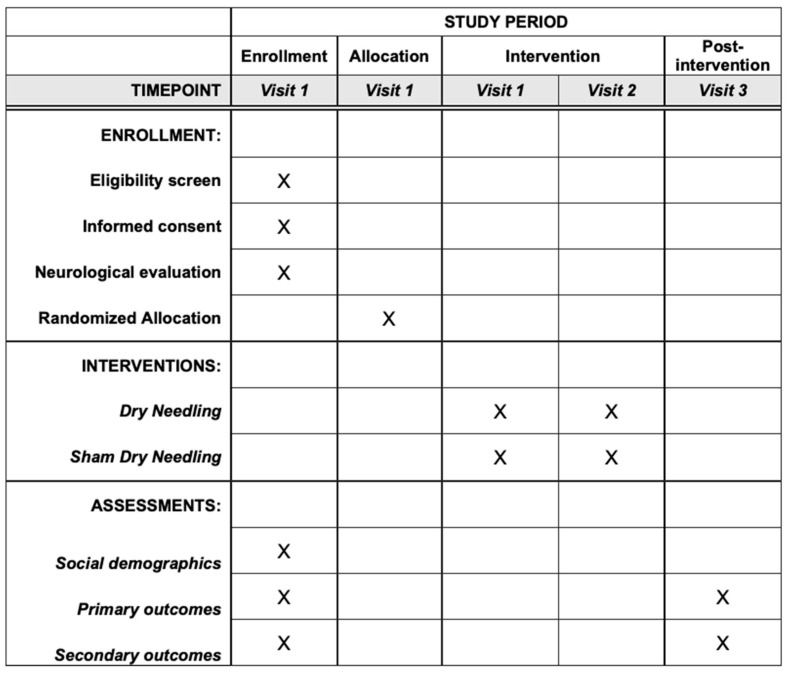
SPIRIT recommended content for the schedule of enrolment, interventions, and assessments.

The flowchart of the study process is shown in Figure 1 and Figure 2. At a first appointment, a neurologist will carry out a complete neurological evaluation of each patient to assess for eligibility. Data collection will be carried out at baseline (first visit) and between 6 and 8 days after the intervention protocol by a researcher (evaluator) who will be blinded to participants’ allocation group. The intervention in both groups will be performed by another senior physiotherapist, who is experienced in the use of DN.

The lead researcher (physical therapist) will get the signed informed consent forms, where the study details will be clearly explained, and the patients will be informed that his/her participation is voluntary so they can withdraw at any moment with no need to provide any specific reason.

### 2.4. Interventions

All patients will continue receiving their usual care during the study protocol. In addition, the DN interventions in both groups will be carried out in the Research and Treatment Unit in Multiple Sclerosis, Dinac Foundation, at Vithas Nisa Hospital, Seville, Spain.

For those in the DN group, the intervention will consist of using deep DN with disposable stainless-steel needles (0.3 mm × 50 mm or 0.3 mm × 40 mm, Agupunt, Barcelona, Spain), depending on the depth of the muscle to be treated. The fast in–fast out Hong technique will be used over the active or latent myofascial trigger points of the RF and GM muscles, following the diagnostic criteria and intervention guidelines previously established [36]. A minimum of 3 to 4 local twitch responses will have to be elicited. The participants’ level of tolerance will be considered, and participants will be able to request to stop the treatment at any moment if unpleasant or very painful sensations appear. The intervention will be performed in the LE with higher spasticity, as expressed by the Modified Ashworth Scale score in the targeted muscles. A single puncture per muscle will be made each session. DN will be performed during 45–60 s for muscle until the local twitch responses disappeared or until the patient can no longer bear the pain. The intervention will last for 2 weeks, 1 session per week.

For the placebo group, a simulation needle procedure using a Dong Bang placebo needle, similar to the Streitberger needle, will be used. These needles appear to be effective placebo techniques in studies using acupuncture [37]. The physical therapist will follow the same treatment protocol than in the DN group. The placebo needles evoke mechanical stimulation without piercing the skin; hence, patients experience a pressure sensation similar to that of a “real” needle.

### 2.5. Outcomes

Clinical assessment of gait performance, spasticity level, electromyographic activity, pain, ROM, and QOL will be reported in the first and third visit (Figure 2). Several outcome measures will be collected to get a better understanding of the treatment effect and because of their extensive use in research on these topics. This will allow us to compare our results with those reported in previous studies. Following the guidelines of the International Classification of Functioning, Disability and Health [38], some of the outcomes will be devoted to investigate functional parameters, such as gait parameters and QOL. The outcome measures and the tools to assess them are detailed below:

#### 2.5.1. Gait Performance

The GaitRite^®^ system will be used to measure several gait parameters (average gait speed, walking cadence, step length, stride length, step width, and percentage of support-oscillation) [39,40]. During the overground walking assessment, participants will walk at preferred speed along an 8.62 m walkway (the length of the tapestry). Data about gait parameters will be collected with motion sensors placed directly in the tapestry.

The two-minutes walking test (2MWT) [41] will be used to assess functional capacity. Given the difficulty that many patients with MS have to walk for 6 min, we propose the 2MWT in the present protocol. The 2MWT is valid in pwMS and provide an efficient and alternative to the 6MWT.

#### 2.5.2. Spasticity

The Modified Ashworth scale (MAS) [42] will be used to assess the spasticity level over the RF and GM muscles. The reliability of the MAS has been demonstrated in LE muscles, e.g., knee extensors and plantar flexors. Thus, this scale is a valid tool for the present protocol. Even though the MAS only measures the passive component of spasticity, the electromyographic evaluation detailed below would provide some information on the spastic co-contraction phenomena during the execution of analytical movements.

#### 2.5.3. Electromyographic Activity

A superficial Electromyography (sEMG) will measure the neuromuscular activity over the RF and GM muscles and their activity during gait. This outcome will be collected with a sEMG device called mDurance [43,44]. The sEMG sensors will be placed in the motor point of the RF and GM muscles according to the recommendations of the SENIAM protocol [45]. To obtain the Maximal Voluntary Contraction [46] for the RF muscle, patients will lie on their back with the leg off the table (hip in extension, and knee in 90° flexion), in the stretch position, and the patient will be asked for a maximum muscle contraction and to keep it for 6 s. For the GM muscle, the patient will be placed lying on his back. With the ankle in dorsal flexion, the patient will be asked to move the ankle to plantar flexion, and to keep the muscle contraction for 6 s. Additionally, the activity of the RF and GM muscles will be monitored during gait, both during the first minute of the 2 MWT and during the GaitRite^®^ examination. In both situations, the Root Mean Square [10] for those muscles will be measured to indirectly quantify muscle fatigue.

#### 2.5.4. Pain

The pressure pain threshold, as the minimal amount of pressure where the sensation of pressure changes to pain, will be collected with a pressure algometer (Psymtec Digital Algometer M3) [47]. The measurement will be performed bilaterally at several spots: (a) over the motor point of the RF muscle and GM muscles; (b) at the head of the splint bone, considered as a control point close to the intervention sites; and (c) distally, over the upper trapezius, as a remote control location.

#### 2.5.5. Range of Movement

The passive hip, knee, and ankle ROM will be assessed using a manual standard goniometer. ROM is a common outcome measure in other studies about the efficacy of DN in different conditions.

#### 2.5.6. Quality of Life

The Multiple Sclerosis International QoL (Musiqol) [48] questionnaire will measure changes in the QOL. This is a multi-dimensional and self-administered questionnaire, and is available in 14 languages, including Spanish. This tool is a disease-specific QOL scale that includes items related to activities of daily living and psychological aspects, among others.

### 2.6. Recruitment, Allocation, and Blinding

The patients will be recruited as they attend their usual consultations with the neurologist, where they will be proposed to take part on the study based on the fulfillment of the established eligibility criteria. Information about the project will be posted on the institutional website where the study will be carried out.

Concerning the allocation concealment procedures, an external researcher will conduct central randomization using computer-generated random numbers (www.randomizer.org), performing a simple randomization. The two lists, DN and placebo groups, comprising a total of 40 patients, will be sent by email to the physical therapist who will perform the intervention, with the randomized numbers assigned to each group. Those numbers will correspond to the recruitment order of each patient, and they will be assigned by the physical therapist in charge of the evaluation, who will remain unaware of the allocation sequence (blinded evaluation).

To ensure blinding, participants will be fitted with a mask so they will not be able to see the type of needle used. Additionally, care providers will not be allowed to access to the room where the intervention will take place, and data analysts will be blinded to participants’ allocation group. All patients will be informed of their group allocation after analyzing the study findings. If real DN shows to be superior to placebo DN, participants in the placebo group will be allowed to receive the real DN protocol.

### 2.7. Data Collection and Management

The data will be entered directly into the computer where the patient’s medical record is stored, which is placed in the facilities where the evaluation will be carried out. This process will guarantee the security of the data. For the exchange of data between researchers for further analysis, an Excel page will be sent, including only the identification number of each patient.

### 2.8. Statistical Methods

#### 2.8.1. Sample Size

The G*Power version 3.1 statistical software (Heinrich-Heine-Universität Düsseldorf) was used to estimate the sample size and statistical power. According to Salom-Moreno et al. [21] the between groups difference in pressure pain sensitivity, assessed with a pressure algometer, was 39.7 ± 79.92 kPa (effect size *d* = 0.49) in the anterior tibialis muscle of the affected side. Furthermore, since this previous study did not use a placebo group as a comparison group, the study of Ghannadi et al. [25] was considered to calculate the sample size according to the MAS, that showed a difference between groups of 0.75 ± 0.82 points (effect size *d* = 0.93). This would require a sample size of at least 38 participants. An alpha level of 0.05 was assumed to perform the analysis. In both cases, the calculated sample size had an 85% power to detect statistically significant and clinically meaningful changes in pressure pain sensitivity and spasticity. Therefore, we considered 40 patients to be an adequate sample size for the proposed study.

#### 2.8.2. Data Analysis

Statistical Analysis will be performed by PASW STATISTIC 18 for Windows. Descriptive statistics will be calculated to show a summarized overview of the data, including frequency counts for categorical variables and central tendency and dispersion measurements for continuous variables. Means, standard deviations (SD) and 95% confidence intervals (CI) will be calculated for continuous variables. The Kolmogorov-Smirnov test will be used to check the normality of the data. Baseline characteristics will be compared between groups to assess the adequacy of the randomization process and analyze the homogeneity of the sample. Independent t-test (normal distribution) or Mann–Whitney U test (non-normal distribution) for continuous data or chi-square tests of independence for categorical data will be used for this purpose. Furthermore, within groups (paired) and between groups (unpaired) pre-post intervention differences will be analyzed using the abovementioned statistical tests, considering the nature of each variable. For all the analyses, an a priori alpha level of 0.05 will be used.

#### 2.8.3. Oversight and Monitoring

The research members (CLM, AGD, JAMM, and AMHR) who conceived the study design and got funding will be part of the trial steering committee. The key role of the chief investigator (CLM) is to coordinate the ongoing project. The trial steering committee will review the progress of the trial and set up the changes and adjustments needed. In case of any serious adverse events during the trial, the lead investigator (CLM) will report them to the Institutional Research Ethics Committee (Comité de Ética de la Investigación Biomédica de Andalucía, CEI Hospitales Universitarios Virgen Macarena—Virgen del Rocío, Seville, Spain) and changes, if needed, will be discussed, and addressed.

During data collection protocol, participants will be asked to report any adverse effects. Upon completion of the study, telephone follow-up will be conducted to check for any possible adverse effects after the last treatment session.

## 3. Discussion

This innovative study will evaluate the impact of DN on gait performance, spasticity level, electromyographic activity, pain, ROM, and QoL in pwMS. Including a sham placebo DN group will allow us to discuss which effects may be related to patient’s expectations and the possible motor behavior adaptations due to this effect. We selected the RF and GM as the targeted muscles for their role in the functionality of the LE in this population. These two muscles are largely responsible for the so-called stiff knee pattern [49,50] caused by an excessive activation of knee extensors during the pre-swing phase [50], along with weakness of the gastrocnemius muscles [49]. This pattern is especially prevalent in pwMS. As regards the treatment protocol, there is some controversy about the number of sessions required to evoke changes after DN interventions, which range from 1 to 3 sessions in most of previous research in this field [23]. In our protocol, we will assess the changes after 2 treatment sessions.

Gait performance and the electromyographic activity of RF and GM muscles have been included as outcomes to detect the electrophysiological changes that may occur at the muscular level, although the specific mechanisms of action of the DN intervention remains controversial [30]. This will allow us to investigate the motor pattern changes. This appears to be an important outcome after using an invasive technique that acts directly over the muscle tissue which could evoke changes in the gait pattern. Thus, the interpretation of the electromyographic parameters by means of maximum voluntary contraction and root mean square may inform us about changes related to muscle force and fatigue. Additionally, functional electromyographic measurements during gait will help us to understand how these changes can influence gait patterns.

One of the strengths of this protocol is the inclusion of the placebo group. The clinical results when using invasive approaches can be influenced by patients’ expectations, as an adaptation associated with the placebo effect. It has been also speculated that some patients could also benefit for these treatments when no real invasive approaches are used. Nevertheless, there is controversy about this issue [27], especially in patients with sensory motor deficits. Recently, the effect of DN was compared with sham DN in a single study in stroke patients [25] that found a decrease in muscle spasticity and improvements in function and gait speed after three sessions. Since stroke is a neurological disorder with different clinical features than MS, a clinical trial comparing real deep DN with sham placebo DN in pwMS is of high interest. The findings of this study may determine if one of the two approaches is superior to the other, which is relevant from a clinical point of view.

DN is a cheap and easy to carry out technique and serious adverse events are rare. Positive findings are expected after this study. If this is the case, DN could be considered as a feasible and useful complementary intervention, within a multimodal approach, to enhance the functional recovery of pwMS during the different clinical stages of the disease.
